# Metabolic Alterations Associated with Atorvastatin/Fenofibric Acid Combination in Patients with Atherogenic Dyslipidaemia: A Randomized Trial for Comparison with Escalated-Dose Atorvastatin

**DOI:** 10.1038/s41598-018-33058-x

**Published:** 2018-10-02

**Authors:** Ji Soo Han, Kyu Kim, Youngae Jung, Jae-Hwan Lee, June Namgung, Hae-Young Lee, Jon Suh, Geum-Sook Hwang, Sang-Hak Lee

**Affiliations:** 1 Integrated Metabolomics Research Group, Western Seoul Center, Korea Basic Science Institute, Seoul, Korea; 2 Division of Cardiology, Department of Internal Medicine, Severance Hospital, Yonsei University College of Medicine, Seoul, Korea; 3 Department of Cardiology in Internal Medicine, School of Medicine, Chungnam National University, Chungnam National University Hospital, Daejeon, Korea; 4 Division of Cardiology, Department of Internal Medicine, Inje University Ilsan Paik Hospital, Goyang, Korea; 5 Department of Internal Medicine, Seoul National University Hospital, Seoul, Korea; 6 Division of Cardiology, Department of Internal Medicine, Soonchunhyang University Hospital, Soonchunhyang University College of Medicine, Bucheon, Korea

## Abstract

In the current study, the metabolic effects of atorvastatin dose escalation versus atorvastatin/fenofibric acid combination were compared using metabolomics analyses. Men and women with combined hyperlipidaemia were initially prescribed atorvastatin (10 mg, ≥4 weeks). Patients who reached low-density lipoprotein-cholesterol targets, but had triglyceride and high-density lipoprotein-cholesterol levels ≥150 mg/dL and <50 mg/dL, respectively, were randomized to receive atorvastatin 20 mg or atorvastatin 10 mg/fenofibric acid 135 mg for 12 weeks. Metabolite profiling of serum was performed and changes in metabolites after drug treatment in the two groups were compared. Analysis was performed using patients’ samples obtained before and after treatment. Of 89 screened patients, 37 who met the inclusion criteria were randomized, and 34 completed the study. Unlike that in the dose-escalation group, distinct clustering of both lipid and aqueous metabolites was observed in the combination group after treatment. Most lipid metabolites of acylglycerols and many of ceramides decreased, while many of sphingomyelins increased in the combination group. Atorvastatin dose escalation modestly decreased lysophosphatidylcholines; however, the effect of combination therapy was variable. Most aqueous metabolites decreased, while l-carnitine remarkably increased in the combination group. In conclusion, the atorvastatin/fenofibric acid combination induced distinct metabolite clustering. Our results provide comprehensive information regarding metabolic changes beyond conventional lipid profiles for this combination therapy.

## Introduction

Fenofibrate, a peroxisome proliferator-activated receptor alpha (PPARα) agonist, is frequently used with statins to treat patients with dyslipidaemia and cardiovascular risk^1,2^. Further, fenofibrate has been reported to have beneficial effects against microangiopathy in diabetic individuals. For example, fenofibrate slowed the progression of diabetic retinopathy^3^ and impairment of renal function^4^. In addition, some recent consensuses recommend the prescription of fenofibrate based on cardiovascular benefits demonstrated in populations with atherogenic dyslipidaemia^5,6^.

A major biological role of PPARα agonist altering lipoprotein metabolism, resulting in enhanced production of apolipoprotein A1, reduced production of very low-density lipoproteins, increased lipolysis of triglycerides, and clearance of low-density lipoproteins^7^. In addition, PPARα agonists are known to modulate a large number of target genes that are involved in lipid transport, acyl-CoA metabolism, β-oxidation, ketogenesis, lipogenesis, lipolysis, lipoprotein metabolism, bile transport, glucose metabolism, amino acid metabolism, and inflammation^8,9^. Furthermore, it has been reported that PPARα activation decreases steatosis and chronic inflammation of liver^10^. Since PPAR agonists, including α isotype, display various metabolic effects, targeting PPARs in metabolic disorders is still a promising approach for metabolic disorders^11^.

Since metabolomics analysis techniques have been introduced, several studies have evaluated metabolic changes accompanying lipid modifying therapy. However, most of these studies examined changes in selected metabolites after a single drug treatment^12,13^.

The aim of the current study was to compare the metabolic effects of two lipid-modifying regimens. We examined whether dose escalation of atorvastatin and atorvastatin/fenofibric acid combination have differential metabolic effects. In addition to conventional lipid parameters, lipid and aqueous metabolites were comprehensively analysed using a metabolomics approach. In real-world clinical practice, patients with atherogenic dyslipidaemia are basically prescribed a statin, and a part of them are additionally prescribed a fibrate. Accordingly, a metabolic comparison of the two approaches (escalation or combination) in patients already taking statins is more helpful from a practical point of view. Our target regimens were determined considering this clinical background.

## Results

### Patient characteristics

A total of 89 patients were initially screened. After a 4-week run-in period, 52 individuals who did not meet the lipid criteria were excluded and 37 patients were randomized (19 to the dose-escalation group and 18 to the combination group). Three participants dropped out during the trial and 34 completed the whole study (Supplementary Fig. S1). The study began in August, 2013 and ended in October 2015. Clinical characteristics of the patients who completed the study are shown in Table 1. The mean age was 63 years; 9 (26%) were females; 11 (32%) were diabetic; and 21 (62%) had coronary artery disease. The mean pre-randomized LDL-C level was 81 mg/dL. Baseline characteristics of the two groups were not different except for high density lipoprotein-cholesterol (HDL-C) levels, which were lower in the combination group. There was no harm or unintended effect in each group.

**Table 1:** Baseline characteristics of the study population whose metabolites were analysed.

	Dose-escalation (n = 18)	Combination (n = 16)	*p*
Age, years	60.5 ± 8.3	64.4 ± 6.5	0.14
Female (%)	4 (22)	5 (31)	0.84
**Medical history (%)**			
Diabetes mellitus	7 (39)	4 (25)	0.62
Hypertension	10 (56)	14 (88)	0.10
Current smoker	7 (39)	4 (25)	0.62
Coronary artery disease	12 (67)	9 (56)	0.21
Body mass index, kg/m^2^	25.8 ± 2.8	25.8 ± 2.3	0.98
**Laboratory values**			
TC, mg/dL	159 ± 24	158 ± 27	0.93
TG, mg/dL	225 (179, 295)	248 (204, 299)	0.67
HDL-C, mg/dL	38.9 ± 4.8	35.1 ± 5.2	0.03
LDL-C, mg/dL	80 ± 20	82 ± 20	0.85
ApoB, mg/dL	89 ± 16	87 ± 17	0.70
ApoA1, mg/dL	129 ± 22	119 ± 16	0.14
ApoB/A1	0.65 (0.61, 0.73)	0.74 (0.66, 0.79)	0.12
**Medications (%)**			
β-blockers	10 (56)	7 (44)	0.73
Calcium channel blockers	6 (33)	10 (63)	0.18
ACE inhibitors or ARBs	10 (56)	13 (81)	0.22
Diuretics	5 (28)	3 (19)	0.83
Antiplatelet agents	15 (83)	10 (63)	0.33

Values are presented as number (%), mean ± standard deviation, or median (interquartile range); TC: total cholesterol; TG: triglyceride; HDL-C: high-density lipoprotein-cholesterol; LDL-C: low-density lipoprotein-cholesterol; apo: apolipoprotein; ACE: angiotensin converting enzyme; ARB: angiotensin receptor blocker.

### Changes in conventional lipid parameters

Changes in conventional lipid parameters are presented in Supplementary Table S2.

### Multivariate analysis of lipid metabolites

**Figure 1:**
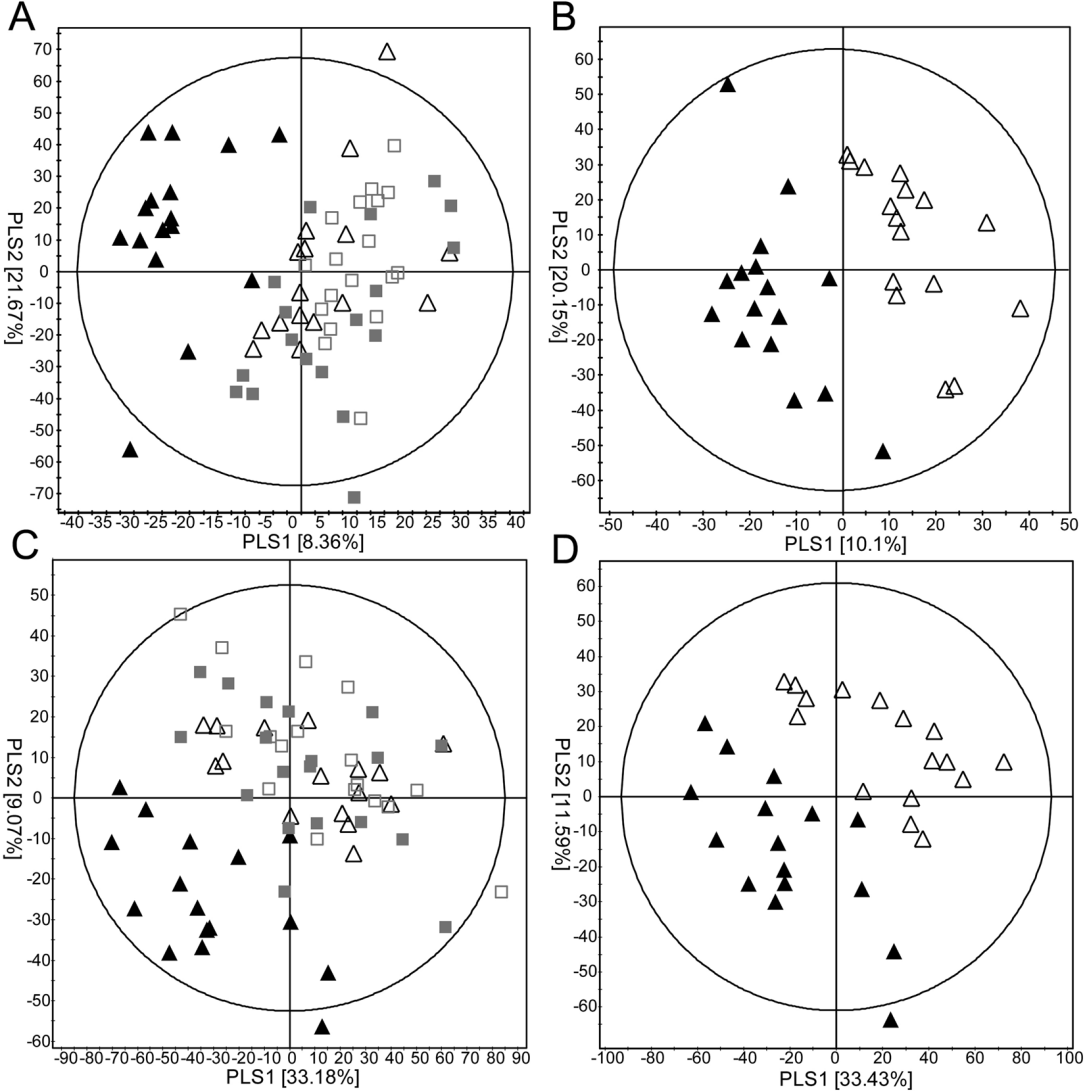
Partial-least-square discrimination analysis (PLS-DA) scatter plots of serum lipid metabolites in the dose-escalation and combination groups. Ultra-performance liquid chromatography quadruple time of flight mass spectrometry (UPLC/QTOF MS) spectra in the positive (**A**,**B**) and negative modes (**C**,**D**) are shown. Data from the dose-escalation and combination groups before and after drug treatment are displayed in (**A**,**C**), whereas data from the combination group only are presented in (**B**,**D**). □: dose-escalation, before; ■: dose-escalation, after; △: combination therapy, before; ▲: combination therapy, after.

To compare the effect of two regimens, multivariate analysis was applied to UPLC/Q-TOF-MS data of the lipid extracts. PCA scatter plots derived from lipid extracts did not show a clear separation between the two groups before or after drug treatment in both positive and negative ESI modes (Supplementary Fig. S2A,B). In contrast, using 3D plots, lipid metabolites from the combination group only after treatment clustered well (Supplementary Fig. S3A,B). QC samples (pooled from every patient sample at consistent intervals) were analysed. QC samples clustered well within each PCA scatter plot, demonstrating reproducibility of the instrument. Scatter plots of lipid metabolites using PLS-DA, a supervised method, showed distinctively discriminated clustering patterns after combination therapy within the positive (R^2^X = 0.299, R^2^Y = 0.274, Q^2^ = 0.081) and negative (R^2^X = 0.199, R^2^Y = 0.340, Q^2^ = 0.071) modes (Fig. 1A,C). A clear separation before and after combination therapy was observed in the PLS-DA scatter plots in both positive (R^2^X = 0.547, R^2^Y = 0.991, Q^2^ = 0.661) and negative (R^2^X = 0.283, R^2^Y = 0.863, Q^2^ = 0.589) modes (Fig. 1B,D). In the PLS-DA plot, each symbol represents the metabolic pattern of each individual. In Fig. 1A,C, plots of combination group after treatment (▲) clustered on the left of PLS1 axis, indicating that combination group after treatment had different metabolic pattern than other groups. Likewise, a clear separation between two groups on the PLS1 axis in Fig. 1B,D indicates metabolic change in combination group after treatment. However, no separation was observed in the PLS-DA plots before and after treatment in the dose-escalation group. These results suggest that appreciable changes in lipid metabolites were associated with the combination rather than dose-escalation treatment.

### Significant changes in lipid metabolites

**Figure 2:**
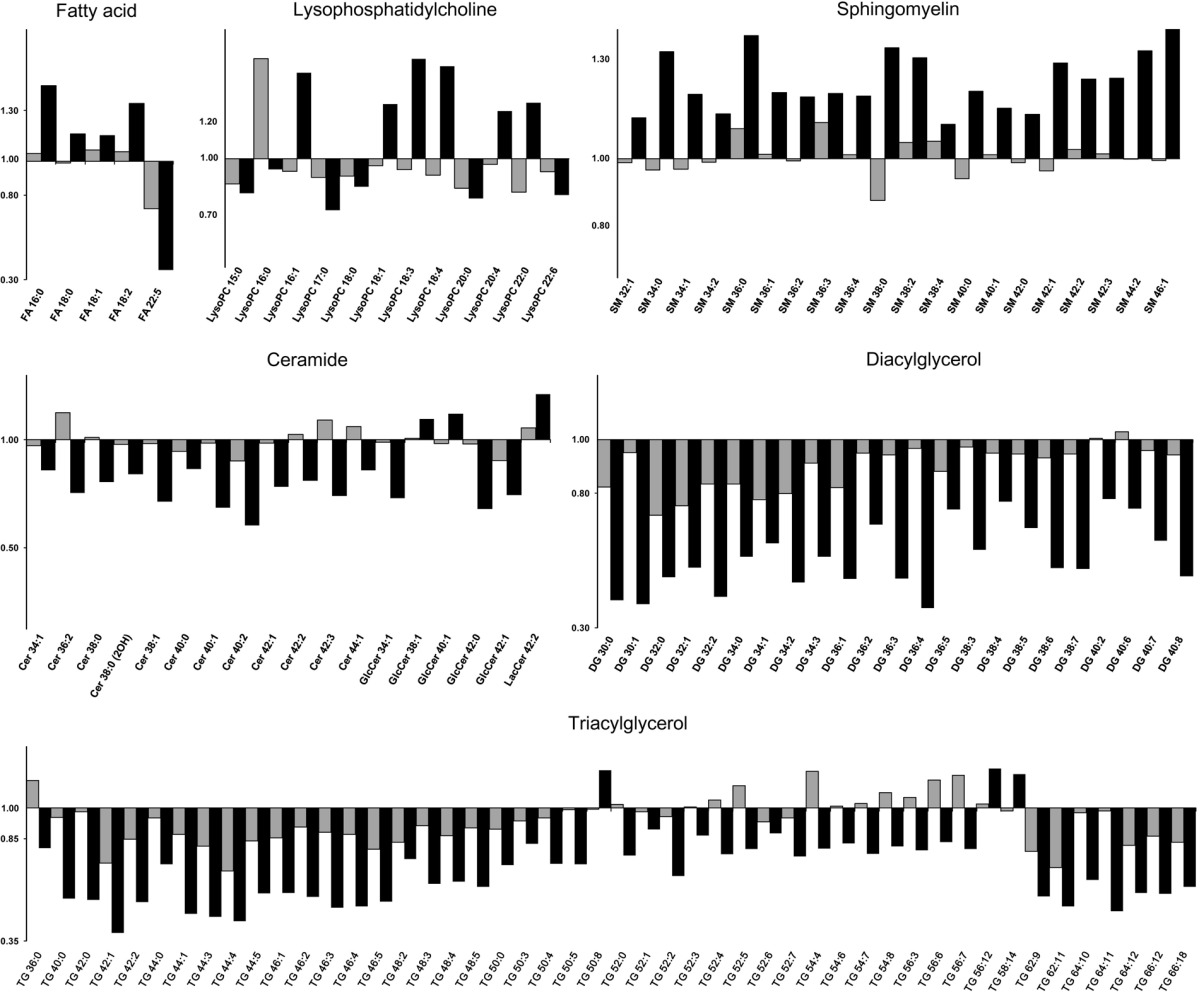
Lipid metabolite changes in the dose-escalation and combination groups. Lipid metabolite data are presented with a bar graph using fold changes after/before treatment. Gray and black bars indicate the escalation and combination groups, respectively. Changes were significant for all metabolites of combination group and nine (FFA 22:5, DG 32:0, DG 34:1, PC 42:11, PI 32:1, PI 34:1, PI 36:4, TG 42:1, and TG 64:10) of dose-escalation group.

To understand and determine the contribution of significant lipid metabolites associated with the changes after combination therapy, we performed a PLS-DA to observe the distinct discriminations in the plot. Significantly different metabolites in the combination group were identified using variable important projection (VIP) >1 and *p* < 0.05 as selection criteria. Selected metabolites are listed according to their categories, including free fatty acids (FFA), acylcarnitines, cholesterol esters, LPCs, lysophosphatidylethanolamines, lysophosphatidylamines, phosphatidylamines, phosphatidylcholines, phosphatidylethanolamines, phosphatidylinositols, sphingomyelins, ceramides, diacylglycerols, and triacylglycerols. The most remarkable lipid metabolite data involved diacylglycerols and triacylglycerols, which predominantly decreased regardless of the degree of chain length or saturation. Diacylglycerols and triacylglycerols decreased further in the combination group. In addition, the level of numerous ceramides decreased, and sphingomyelin levels increased after combination therapy (Fig. 2 and Supplementary Table S3); however, the changes in some ceramides and sphingomyelins did not follow the overall trend observed in the respective categories. (Fig. 2). Further, FFAs increased in the combination group (except FFA 22:5), while the fold change in FFAs showed a reverse pattern in the escalation group. The escalation group showed a modest decrease in LPCs; however, changes in LPC after combination therapy were variable (Supplementary Table S3).

### Multivariate analysis of aqueous metabolites

**Figure 3:**
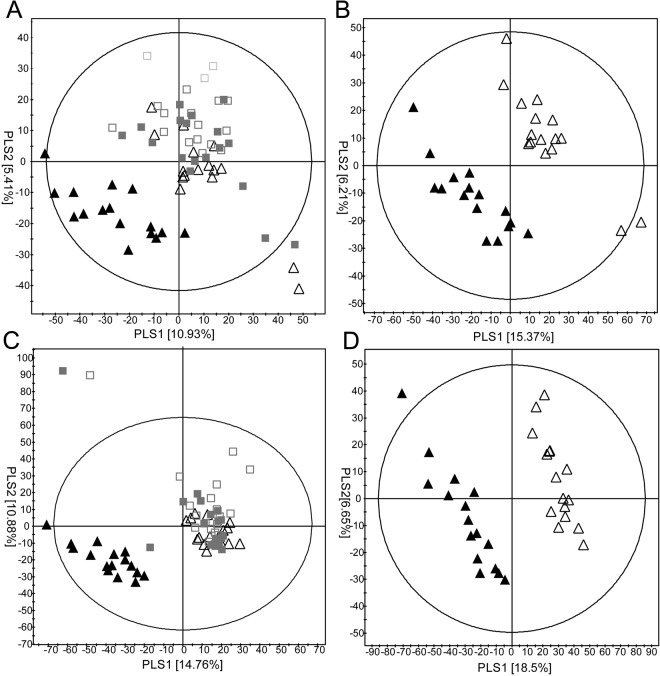
Partial-least square discriminant analysis (PLS-DA) scatter plots of serum aqueous metabolites in the dose-escalation and combination groups. Ultra-performance liquid chromatography quadruple time of flight mass spectrometry (UPLC/QTOF MS) spectra in the positive (**A**,**B**) and negative modes (**C**,**D**) are shown. Data from the dose-escalation and combination groups before and after drug treatment are displayed in **A**,**C**, whereas data from the combination group only are presented in (**B**,**D**). □: dose-escalation, before; ■: dose-escalation, after; △: combination therapy, before; ▲: combination therapy, after.

Similar to the lipid metabolite data, PCA scatter plots derived from aqueous extracts did not show distinct separation patterns before and after drug treatment in either group in both positive and negative ESI modes (Supplementary Fig. S2B,D). When the PCA scatter plots were visualized in 3D, aqueous metabolites seemed to cluster after combination therapy (data not shown). To exclude the possibility of any artificial influence on reliability and precision, we also analysed QC samples; results showed that reproducibility of the instrument was respectable. No significant changes in the amount or class of metabolites were observed, except for the comparison data before and after combination therapy (data not shown). PLS-DA models of aqueous metabolites showed unique clustering patterns in the positive (R^2^X = 0.163, R^2^Y = 0.318, Q^2^ = 0.146) and negative (R^2^X = 0.256, R^2^Y = 0.313, Q^2^ = 0.154) modes after combination therapy only (Fig. 3A,C). We compared metabolites before and after each treatment to figure out differential changes. PLS-DA plots of the combination group showed noticeable separations before and after treatment both in the positive (R^2^X = 0.216, R^2^Y = 0.933, Q^2^ = 0.587) and negative (R^2^X = 0.368, R^2^Y = 0.996, Q^2^ = 0.885) modes (Fig. 3B,D), whereas similar results were not observed for the escalation group.

### Changes in significant aqueous metabolites and acylcarnitine quantitative data

**Figure 4:**
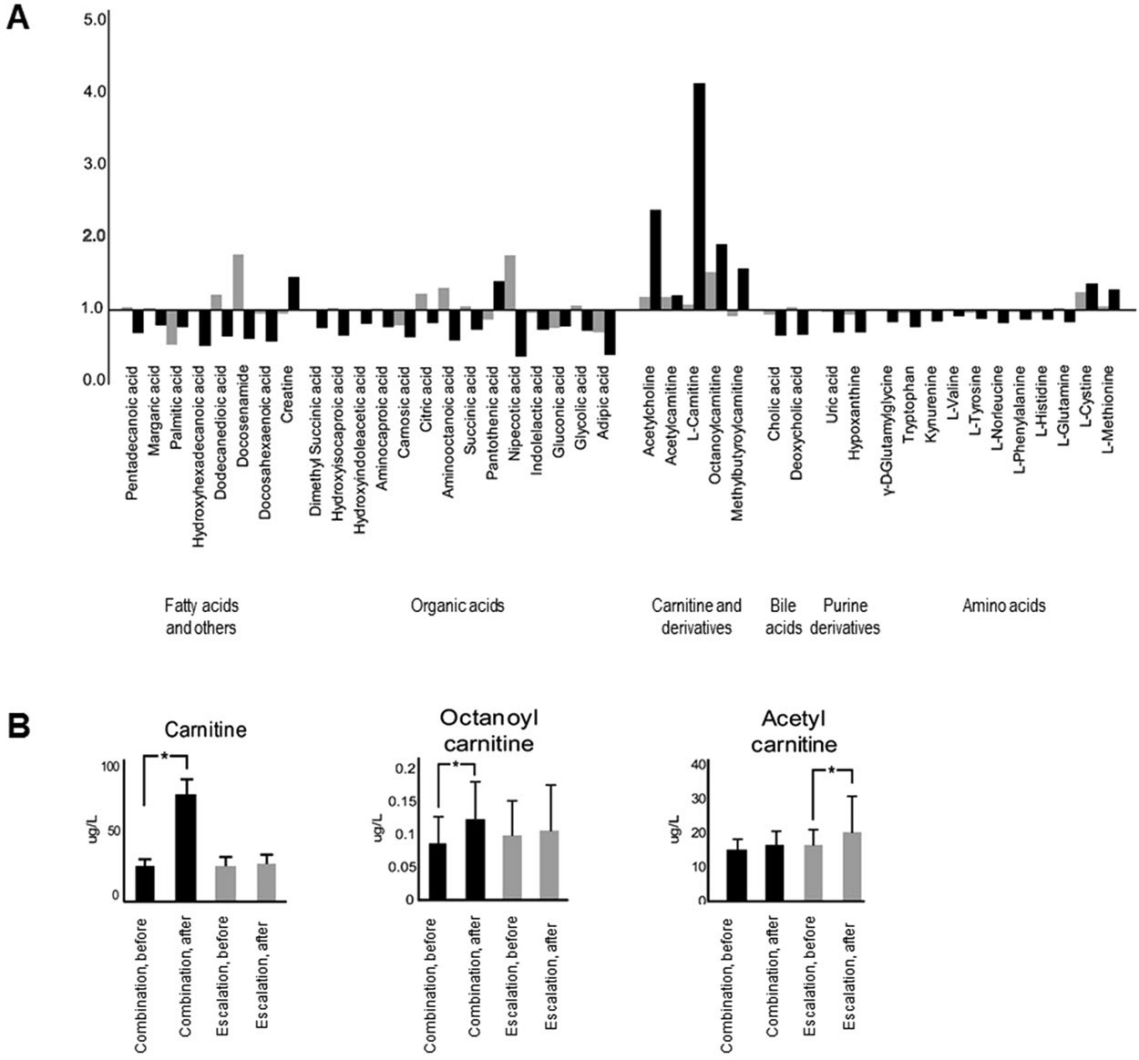
Aqueous metabolite changes in the dose-escalation and combination groups and quantitative analysis of carnitine and its derivatives in serum. Aqueous metabolite data are presented with a bar graph using fold changes after/before treatment (**A**), and the quantitative analysis data of carnitine and acylcarnitine (**B**) is shown. Changes were significant for all metabolites of combination group and one (acetyl carnitine) of dose-escalation group.

Based on the same criteria used to select lipid metabolites, 44 aqueous metabolites were identified as significantly changed after combination therapy. The metabolites are presented according to the following categories: amino acids, organic acids, fatty acids, bile acids, carnitine and its derivatives, and purine and other metabolites. Most of the aqueous metabolites decreased; however, carnitine and acylcarnitine increased after combination therapy (Fig. 4A and Supplementary Table S4). To confirm the remarkable changes in carnitine and its derivatives, we analysed serum from the study patients using Triple Quad mass spectrometry. Three metabolites (carnitine, acetylcarnitine, and octanoylcarnitine) were analysed quantitatively. QC samples were consistently analysed to determine reproducibility. Calibration curves and each sample were analysed with three replicates. Each calibration curve for the quantitatively analysed metabolites showed 99.9% reliability (Supplementary Table 1). The amount of carnitine in serum from patients increased almost 3-fold after combination therapy, while acetylcarnitine and octanoylcarnitine increased 1.2- and 1.8-fold, respectively, after combination therapy (Fig. 4B).

## Discussion

In the present study, the comparison was mainly of the added effect of fenofibric acid on atorvastatin versus dose-escalation of atorvastation. The major findings of this study include: (1) distinct clustering of both lipid and aqueous metabolites occurred after treatment in the atorvastatin/fenofibric acid combination group, whereas this phenomenon did not occur in the atorvastatin dose escalation group; (2) most lipid acylglycerol metabolites and many ceramides decreased in the combination group, while numerous sphingomyelins increased; (3) LPCs modestly decreased in the atorvastatin dose escalation group, whereas the effect of combination therapy on LPCs was variable; (4) most aqueous metabolites decreased in the combination group, while l-carnitine remarkably increased; and (5) changes in cholesterol and triglycerides differed between treatment groups, whereas changes in the apolipoprotein B/A1 (apoB/A1) ratio were similar between the two groups. These results provide comprehensive information regarding metabolic changes beyond conventional lipid profiles for statin/fenofibric acid combination therapy.

The concentration of ceramide metabolites tended to decrease after treatment in the combination group. This is consistent with a very recent report by Croyal *et al*.^14^, in which a global decrease in ceramides was shown after fenofibrate therapy using FIELD trial samples. The reduction in ceramides was possibly related to changes in synthesis in the circulation or in the liver where fenofibrate could affect rate-limiting steps of ceramide synthesis. Although it is not certain, the reduction in ceramides could be indirectly attributable to the carrier effect of very low-density lipoprotein as modulated by fenofibrate. We observed an increasing trend in sphingomyelin metabolites after combination therapy. To date, the effect of fibrates on plasma sphingomyelin is not well known. Some studies have demonstrated that fenofibrate induces higher sphingomyelin on high-density lipoproteins in rabbits^15^ and humans^16^. Fenofibrate treatment enhanced the functional capacity of high-density lipoproteins in the former study, and the increase in sphingomyelin is a potential mechanism underlying the beneficial effect.

We found that the effects of the combination therapy on LPCs were variable; however, they were modestly decreased by higher dose of statin. LPC is produced from phosphatidylcholine of lipoproteins or cell membranes through the action of phospholipase A_2_, which is known to play a role in diverse biological processes including anti-sepsis^17^. In a prior FIELD substudy, HDL lipidomic profiles associated with fenofibrate treatment were investigated. The authors found that LPC was diminished on HDL in their analysis^13^. We did not find similarly clear tendency of LPC after combination therapy. However, we cannot directly compare our results with those of above-mentioned study, since we analyzed LPC in serum rather than in HDL. In addition, phospholipase A_2_ has been reported to have both pro- and anti-atherogenic properties^18^. Among LPCs assayed in humans, LPC 16:0, 18:0, and 18:1 were shown to be associated with inflammation in atherosclerotic plaques^19^. However, clear differential changes in these species of LPC were not observed between the two regimens in the present study.

Of note, plasma l-carnitine clearly increased in the group receiving the combination therapy in our study. A previous study showed that activation of PPARα results in hepatic carnitine accumulation in mice. This phenomenon was mediated by means of enhanced carnitine biosynthesis^20^ and hepatic carnitine import^21^. Conversely, PPARα-knockout mice have 40–50% lower plasma and tissue levels of free carnitine^22^. Another study revealed that PPAR agonism with clofibrate increased intestinal carnitine absorption in rats^23^. Collectively, the above-mentioned studies may partly explain the elevation in l-carnitine after combination therapy in our study. Plasma l-carnitine levels have been reported to be predictive of greater cardiovascular risk. However, that finding was valid only in subjects with concurrently high trimethylamine-*N*-oxide (TMAO) levels^24^. It is not evident whether the increase in l-carnitine after combination therapy in our study has clinical relevance, attributable to a lack of TMAO data in this study. Dietary TMAO reportedly modulates cholesterol and sterol metabolism and suppresses reverse cholesterol transport^24^. In contrast, another study demonstrated that fenofibrate increases reverse cholesterol transport^25^. Furthermore, studies have revealed the beneficial properties of l-carnitine consumption or fish rich in TMAO, which contradicts studies reporting the harmful effects of l-carnitine^26^. Currently, caution is needed when interpreting data showing a rise in l-carnitine by regimen, including fenofibric acid, in terms of cardiovascular risk.

Not surprisingly, TG reduction and elevation of HDL-C were greater in combination group. The changes in the apoB/A1 ratio were comparable between the two groups. Rosenson *et al*.^27^ compared rosuvastatin and rosuvastatin/fenofibric acid at multiple doses and reported that the LDL-C lowering effects were greater in a high-dose statin group than in a low-dose statin/fenofibric acid group. Ballantyne *et al*.^28^ reported similar results in patients who received fenofibric acid in addition to statin therapy. In our current study, changes of LDL-C in dose-escalation group were quite variable, although they were significantly different from those in combination group. Determining the effects of a treatment regimen that affects multiple lipid parameters using measurements of cholesterol alone is inadequate. Instead, indexes, such as the apoB/A1 ratio, which reflect simultaneous changes in multiple lipoproteins^1^ and cardiovascular risk^29^ may be a more appropriate indicator of the efficacy of our treatment regimens. However, we did not further investigate the findings of conventional lipid parameters, since their comparison was not the primary purpose of our study.

Our study has potential limitations. Although prescribing statins in drug-naïve patients can cause specific metabolic changes, the effects of escalating statin dose in patients with ongoing therapy may be less dramatic. We compared metabolic changes after using two different regimens in patients with ongoing lower-dose statin therapy. Thus, less distinct clustering of metabolites after a dose-escalation of statins rather than combination therapy does not necessarily indicate that the former regimen has a smaller total metabolic influence. Our study provides useful information for adopting additional lipid modification policies. However, clinicians should consider the above-mentioned context.

Taken together, these data show that the statin/fenofibric acid combination induced a more distinct clustering of lipid and aqueous metabolites than the dose escalation of atorvastatin. Most lipid metabolites, including acylglycerols and ceramides, and aqueous metabolites decreased, whereas many sphingomyelins and l-carnitine increased after the combination therapy. Our study provides comprehensive information regarding metabolic changes beyond conventional lipid profiles after the statin/fenofibric acid combination therapy.

## Methods

### Study population

We initially screened men and women aged ≥20 years with triglyceride (TG) levels ranging from 150 to 499 mg/dL and low-density lipoprotein-cholesterol (LDL-C) levels requiring lipid-lowering therapy according to the 2004 National Cholesterol Education Program Adult Treatment Panel III guidelines^30^. After lifestyle modification and pharmacologic treatment with atorvastatin 10 mg for more than 4 weeks, individuals who met all the following criteria were enrolled: TG levels 150–499 mg/dL; HDL-C <50 mg/dL; and LDL-C levels <100 mg/dL in patients with coronary heart disease or its equivalent, including diabetes mellitus, or LDL-C levels <130 mg/dL in all other patients. Criteria for exclusion included a history of cerebrovascular or cardiovascular events in the past 3 months, uncontrolled hypertension (systolic ≥180 mmHg or diastolic ≥110 mmHg), uncontrolled diabetes mellitus (haemoglobin A1c levels >9%), serum creatinine or transaminase >2× the upper limit of normal, gall bladder disease, thyroid dysfunction, heavy alcohol drinking, infection, acute or chronic inflammatory disease, cancer, pregnant or breast feeding women, history of adverse events associated with test drugs, or refusal of enrolment. There was no sufficient data for sample size calculation. We referred to best available studies similar to our design and decided to enroll at least 12 to 17 subjects in each group. All participants provided written informed consent.

### Study design

This study was a sub-study of a main trial, and we applied the protocol used in the main study (ClinicalTrials.gov number, NCT 01974297, registered 01/11/2013). This sub-study was a 12-week, randomized, open-label, multicentre study conducted at five sites in Korea. The study protocol conforms to ethical guidelines of the 1975 Declaration of Helsinki and was approved by the institutional review board (IRB) at each centre: IRB, Clinical Trial Center Severance Hospital, Seoul, Korea, IRB of Chungnam National University Hospital, Daejeon, Korea, IRB, Inje University Ilsan Paik Hospital, Goyang, Korea, IRB, Seoul National University Hospital Biomedical Research Institute, Seoul, Korea and IRB, Soonchunhyang University Hospital, Bucheon, Korea.

At the screening visit, patients were interviewed about their medical history and underwent a physical examination and laboratory assessment. Individuals who met the inclusion criteria after treatment with atorvastatin 10 mg were randomly assigned to receive one of two regimens: atorvastatin 20 mg (Newvast; Hanmi Pharm Co. Ltd., Seoul, Korea) (dose-escalation group) or atorvastatin 10 mg (Newvast) plus fenofibric acid 135 mg (Fenocid; Hanmi Pharm Co. Ltd.) (combination group). Sequentially numbered containers provided by AMD Korea (Seoul, Korea) were used for random allocation. All subjects in the five centers were assigned by block randomization. The study patients were instructed to take medications in the morning. Those in combination group took both components at the same time. Participants were followed up at the end of weeks 6 and 12 for tolerability and efficacy assessments.

Fasting blood samples were collected at randomization and at the end of week 12 of drug treatment. Laboratory values including lipid profiles were measured at these time points. Samples were analysed within 4 h of collection by a local laboratory that was certified by the Korean Society of Laboratory Medicine.

### Sample extraction

Serum samples (50 *μ*L) were transferred to a 1.5-mL tube and vortexed with 550 *μ*L CHCl_3_:MeOH = 2:1 for 1 min. After adding 100 *μ*L water, the samples were again vortexed for 1 min. The mixture was incubated at 4 °C for 10 min and centrifuged for 10 min at 13,000 × *g* and 4 °C. The supernatant (aqueous extract) was transferred into a 1.5-mL Eppendorf tube and dried using a vacuum concentrator. The solution under pellet (lipid extract) was transferred to a 1.5-mL Eppendorf tube and evaporated under a stream of nitrogen. For the ultra-performance liquid chromatography quadruple time of flight mass spectrometry (UPLC/Q-TOF-MS) analyses, aqueous extracts were diluted with an acetonitrile:water mixture (3:1, v/v) and lipid extracts were diluted with an isopropanol:acetonitrile:water mixture (2:1:1, v/v/v) and transferred into vials for analysis.

### UPLC/Q-TOF-MS analysis for lipid and aqueous metabolites

Liquid chromatography-electrospray ionization–tandem MS analyses of lipid extracts and aqueous extracts were performed on a triple TOF™ 5600 MS/MS system (Sciex, Concord, ON, Canada) combined with a UPLC system (Waters, Milford, MA, USA). Lipid extracts were separated on an Acquity UPLC BEH C18 column (2.1 × 100 mm with 1.7-*μ*m particles; Waters), whereas aqueous extracts were separated on a SeQuant^®^ ZIC^®^ hydrophilic interaction liquid chromatography column (2.1 × 100 mm with 3.5-*μ*m particles) (Merck Korea, Incheon, Korea).

The binary gradient system for lipid extracts comprised 10 mM ammonium acetate in an acetonitrile:water mixture (40:60, v/v; solvent A) and 10 mM ammonium acetate in an acetonitrile:isopropanol mixture (10:90, v/v; solvent B); for aqueous extracts, 0.1% formic acid with 10 mM ammonium acetate in an acetonitrile:water mixture (95:5, v/v; solvent A) and 0.1% formic acid with 10 mM ammonium acetate in an acetonitrile:water mixture (50:50, v/v; solvent B) were used. The sample injection volume was 5 *μ*L, and the partial loop mode for both positive and negative ionization polarity modes was used. MS with chromatography is used to detect metabolites that are positively or negatively charged by their ionization characteristics. Metabolites, such as fatty acids and organic acids, are covered in negative modes, while triacylglycerols and diacylglycerols are covered in positive modes.Pooled quality control (QC) samples were measured for data reproducibility.

### Quantitative analysis of metabolites

To perform quantitative analysis, ultra-high performance liquid chromatography triple quadrupole mass spectrometry analyses of aqueous extracts, including carnitine, acetyl-carnitine, and ocatonyl-carnitine, were performed on an Agilent 1290 Infinity LC and an Agilent 6495 Triple Quadrupole MS system equipped with an Agilent jet stream ESI source (Agilent Technologies, Palo Alto, CA, USA). Separations and the binary gradient system were identical to the UPLC/Q-TOF-MS analysis of the aqueous extracts. The column effluent was introduced into a triple quadrupole mass detector operating in a positive or negative ESI mode. Samples were analysed via the simple reaction monitoring mode for transition of the parent ion to the product ion. Characteristics of multiple reaction monitoring (MRM), calibration curve, and accuracy are shown in Supplementary Table 1.

### Pre-processing of lipid and aqueous UPLC-Q/TOF-MS data

UPLC/Q-TOF-MS spectral data were analysed using MarkerView^TM^ (Sciex, Concord, ON, Canada), which was used to find peaks, perform the alignment, and generate peak tables of *m/z* and retention times. Spectra were normalized to the total spectral area. To identify reliable peaks and decrease instrumental bias, features with coefficients of variation below 20 in quality control samples were selected. Lipids were putatively identified by comparing the experimental data against various lipid metabolite databases, including LIPID MAPS (www.lipidmaps.org), Metlin (metlin.scripps.edu), and Human Metabolome (www.hmdb.ca) databases. Isotope pattern matching and fragment patterns (MS/MS spectra) were similarly used to identify lipid metabolites. Lysophosphatidylcholine (LPC), phosphatidylcholine, and sphingomyelin species showed distinct fragmentation patterns, including an abundant product ion of *m/z* 184 in the positive ESI mode tandem mass spectra. Neutral loss fragments of m/z 141 were used to identify lysophosphatidylethanolamine and phosphatidylethanolamine. Ceramide was analysed by means of neutral loss fragments of 277, 185, and 264. Peak findings, as well as alignment and filtering of raw data were performed using MarkerView Version 1.2.1.1 (Sciex, Concord, ON, Canada).

Data collection parameters in peak findings were set as follows: mass range, *m/z* 50–1500; retention time range, 0.2–21 (18 for aqueous MS data) min; subtraction offset, 5 scans; subtraction multiplier, 1.5; minimum spectral peak width, 1 ppm; and retention time peak width, 5 scans. For peak alignment, retention time and mass tolerances were set at 0.1 min and 10 ppm, respectively. The intensity threshold for peak filtering was set at 10, and peaks detected in fewer than five samples were removed. Peak area matrixes were normalized by total sum area normalization to remove systematic variation among the samples.

Two processes were used to decrease instrumental bias and identify reliable peaks: background peak ions in raw data were eliminated using a ratio of peak QC intensity to blank intensity (QC/blank >3), and peaks exhibiting a large variation in QC were eliminated (coefficient of variation (CV) >20%). All variables were tentatively identified based on free accessible metabolite databases such as Mass Bank (http://www.massbank.jp), Metlin (http://metlin.scripps.edu/), and LIPID MAPS (http://www.lipidmaps.org) and were compared to analysed standards.

For quantification analysis, calibration curves were constructed by plotting the peak area/internal standard peak ratio versus the concentration of each sample using MassHunter quantitative analysis for Triple Quad (Agilent Technologies).

### Statistical analysis

Clinical and laboratory variables were compared using the student’s *t* test or chi-square test. A paired *t* test was used to compare the parameters before and after drug treatment. For the variables showing skewed distribution, the Wilcoxon signed-rank test for the median was used. All the analyses used two-tailed tests with a significance level of 0.05. Statistics for the social sciences version 17.0 (SPSS Inc., Chicago, IL, USA) was used for the analyses. For the UPLC-Q/TOF-MS data, chemometric methods were used to characterize and visualize differences and similarities among the different groups and samples. Principal component analysis (PCA) is an unsupervised method that analyses samples in the absence of information about the samples. Prior to PCA, all variables obtained from UPLC-MS data sets were scaled to unit variance (UV). Partial least squares-discriminant analysis (PLS-DA) was used to maximize class discrimination. PLS finds a linear regression model by projecting the predicted and observed variables to a new space. X and Y data are projected to new spaces, and PLS-DA is a variant method used when Y variable is categorical. The primary goal of PLS is to identify class differences from a multivariate dataset. A class refers to biologically relevant classification, such as humans treated with specific drug^31^. For example, metabolomics studies using this statistical method were performed to determine metabolite levels in bone^32^, heart, and serum of mice^33^, and aortic tissues of humans^34^. PLS-DA regression models are interpreted by R and Q square values. R value indicates fitness of model, and a value close to one means good fitness. Q value indicates goodness of prediction and a value over 0.5 means good prediction. PCA and PLS-DA were conducted using SIMCA-P+ (ver. 12.0; Umetrics, Umea, Sweden). VIP score is the projection of each variable, and it is also the selection criterion for important variable in a PLS model. Variables with scores over or close to one are considered important in a given model.

## Electronic supplementary material

Supplementary information
