# Supplementary material for: Metabolic Alterations Associated with Atorvastatin/Fenofibric Acid Combination in Patients with Atherogenic Dyslipidaemia: A Randomized Trial for Comparison with Escalated-Dose Atorvastatin
Source: Sci Rep. 2018 Oct 2;8:14642. doi: 10.1038/s41598-018-33058-x (PMC6168550; doi:10.1038/s41598-018-33058-x)
Supplement: Supplementary file 1 — Supplementary information [file 41598_2018_33058_MOESM1_ESM.docx]

**Metabolic Alterations Associated with Atorvastatin/Fenofibric Acid Combination in Patients with Atherogenic Dyslipidaemia: A Randomized Trial for Comparison with Escalated-Dose Atorvastatin**

Ji Soo Han, Kyu Kim, Youngae Jung, Jae-Hwan Lee, June Namgung, Hae-Young Lee, Jon Suh, Geum-Sook Hwang, Sang-Hak Lee

**Supplementary Fig. S1. Study flowchart.**


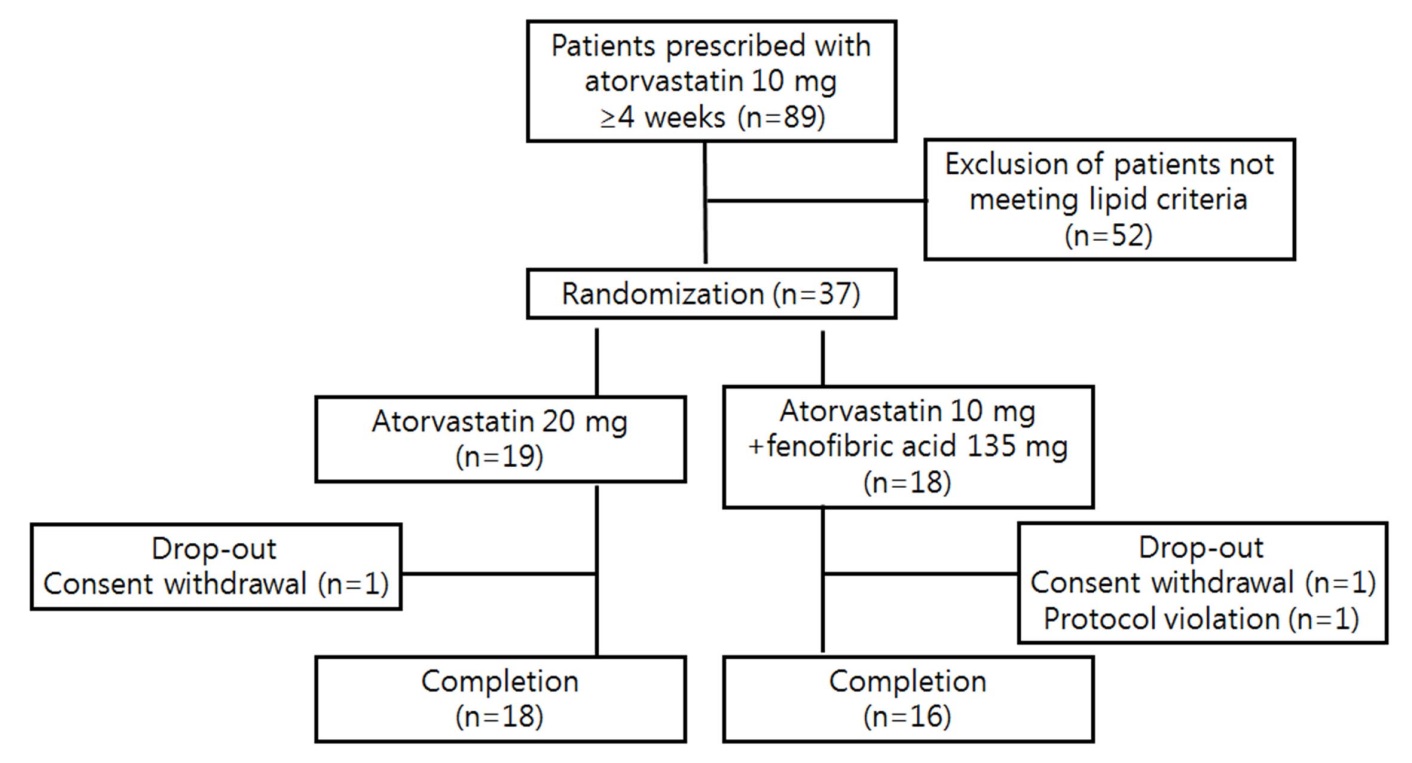


**Supplementary Fig. S2. Principal component analysis (PCA) scatter plots of lipid and aqueous metabolites in dose-escalation and combination groups.** PCA scatter plots obtained from ultra-performance liquid chromatography quadruple time of flight mass spectrometry (UPLC/QTOF MS) spectra of lipid extracts for global analysis are presented in a positive mode (A) and a negative mode (B). The aqueous extract plots are shown in positive mode (C) and negative mode (D).

◇: dose-escalation, before; ◆: dose-escalation, after; △: combination, before; ▲: combination, after

**
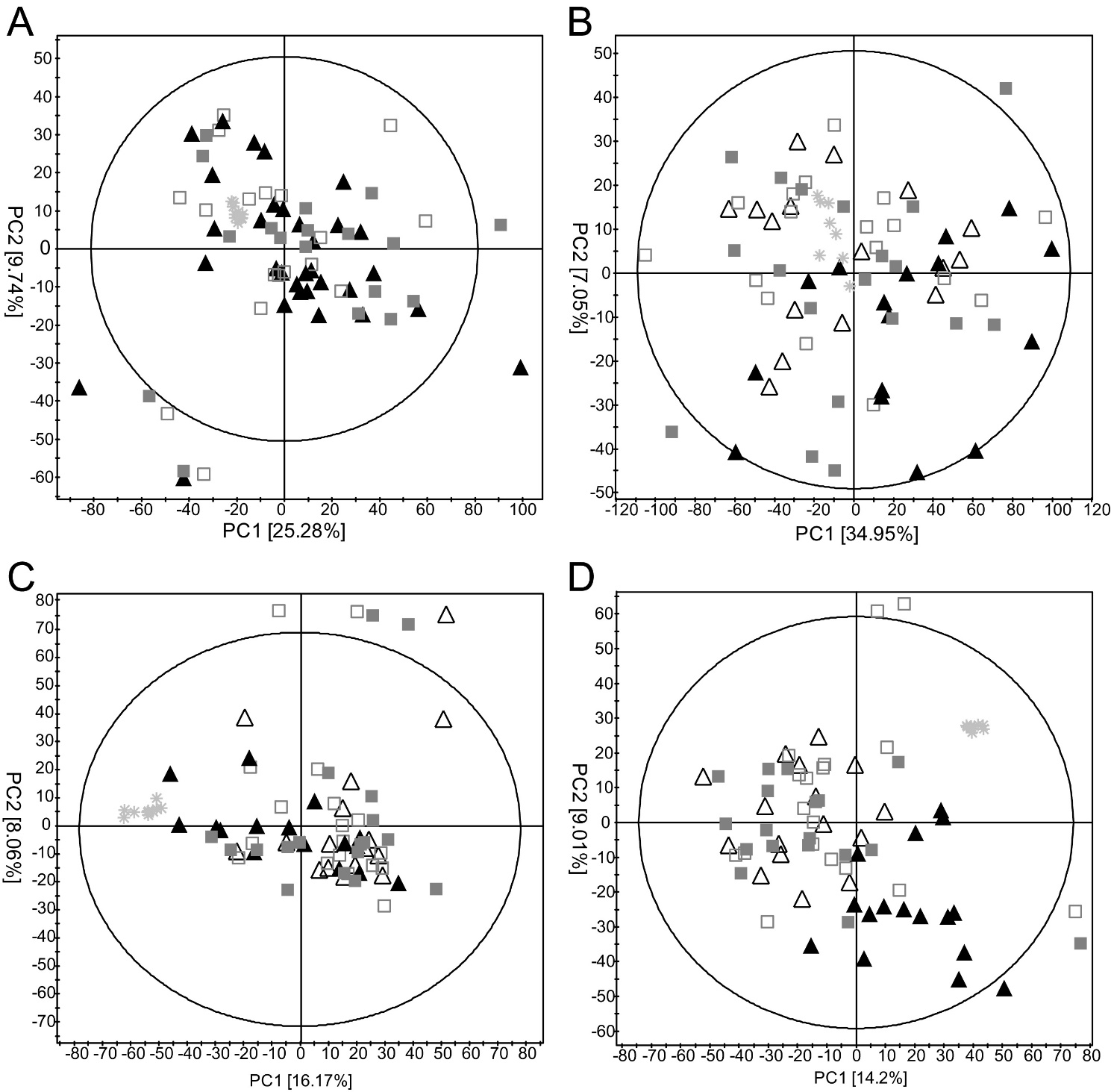
**

**Supplementary Fig. S3. Principal component analysis (PCA) 3D scatter plots of metabolites in dose-escalation and combination groups.** PCA scatter plots obtained from ultra-performance liquid chromatography quadruple time of flight mass spectrometry (UPLC/QTOF MS) spectra of lipid extracts for global analysis are presented in positive mode (A) and negative mode (B).

●: dose-escalation, before; ●: dose-escalation, after; ●: combination, before; ●: combination, after

**
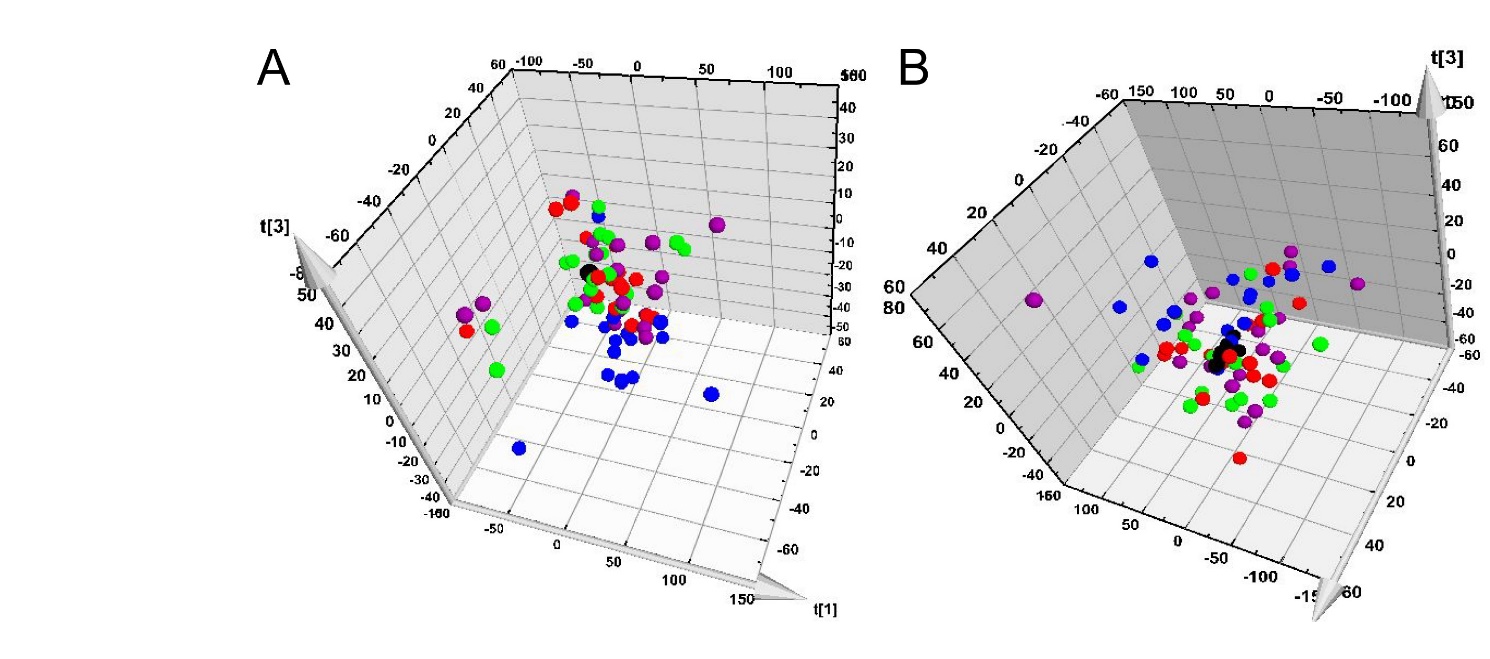
**

**Supplementary Table S1. Multiple reaction monitoring (MRM) transitions of carnitine.**

| **Metabolite** | **m/z** | **Product ion** | **Ion mode** | **Collision**  **energy** | **Mode** | **Calibration curve** | **Accuracy** |
| --- | --- | --- | --- | --- | --- | --- | --- |
| Carnitine | 162.1109 | 85 | Quant | 20 | positive | Y=0.0131638*X-0.04836 | 99.96% |
|  |  | 60 | Qual | 20 | positive |  |  |
| IS_carnitine | 165 | 85 | Qual | 24 | positive |  |  |
| Acetyl  carnitine | 204.1223 | 85 | Quant | 18 | positive | Y=0.023995*X  -0.012850 | 99.95% |
|  |  | 60 | Qual | 20 | positive |  |  |
| IS_acetyl  carnitine | 207 | 85 | Qual | 18 | positive |  |  |
| Octanoyl  carnitine | 305.2475 | 229 | Qual | 8 | positive | Y=2.203078*X  +0.302148 | 99.90% |
|  |  | 85 | Quant | 12 | positive |  |  |
| IS_octanoyl  carnitine | 291 | 85 | Qual | 20 | positive |  |  |

**Supplementary Table S2. Changes in lipid parameters after drug treatment.**

|  |  | Dose-escalation (n=18) | Combination (n=16) | *p*^a^ |
| --- | --- | --- | --- | --- |
| TC, mg/dL | Before | 159 ± 24 | 158 ± 27 | 0.93 |
|  | After | 140 ± 21 | 165 ± 31 | 0.02 |
|  | % change | -11.5 ± 13.3 | 3.2 ± 12.2 | 0.002 |
|  | p^b^ | 0.02 | 0.65 |  |
| TG, mg/dL | Before | 225 (179, 295) | 248 (204, 299) | 0.67 |
|  | After | 196 (133, 273) | 123 (103, 148) | 0.002 |
|  | % change | -14.8 ± 34.7 | -47.8 ± 19.9 | 0.002 |
|  | p^b^ | 0.11 | <0.001 |  |
| HDL-C, mg/dL | Before | 38.9 ± 4.8 | 35.1 ± 5.2 | 0.03 |
|  | After | 39.8 ± 8.9 | 44.1 ± 10.7 | 0.21 |
|  | % change | 1.5 ± 14.5 | 24.7 ± 24.2 | 0.003 |
|  | p^b^ | 0.73 | 0.007 |  |
| LDL-C, mg/dL | Before | 80 ± 20 | 82 ± 20 | 0.85 |
|  | After | 72 ± 16 | 100 ± 22 | <0.001 |
|  | % change | -4.4 ± 38.6 | 26.2 ± 32.3 | 0.02 |
|  | p^b^ | 0.21 | 0.02 |  |
| ApoB | Before | 89 ± 16 | 87 ±17 | 0.70 |
|  | After | 76 ±15 | 87 ± 18 | 0.06 |
|  | % change | -13.2 ± 13.8 | 1.4 ± 12.7 | 0.003 |
|  | p^b^ | 0.02 | 0.90 |  |
| ApoA1 | Before | 129 ± 22 | 119 ± 16 | 0.14 |
|  | After | 126 ± 27 | 134 ± 29 | 0.54 |
|  | % change | -3.0 ± 11.1 | 12.3 ± 16.8 | 0.003 |
|  | p^b^ | 0.67 | 0.08 |  |
| ApoB/A1 | Before | 0.65 (0.63, 0.78) | 0.75 (0.66, 0.79) | 0.12 |
|  | After | 0.59 (0.48, 0.76) | 0.63 (0.57, 0.73) | 0.35 |
|  | % change | -9.8 ± 14.9 | -8.0 ± 17.1 | 0.75 |
|  | p^b^ | 0.24 | 0.27 |  |

Values are presented as number (%), mean±standard deviation, or median (interquartile range); TC: total cholesterol; TG: triglyceride; HDL-C: high-density lipoprotein-cholesterol; LDL-C: low-density lipoprotein-cholesterol; Apo: apolipoprotein; *p*^a^: comparison between groups; *p*^b^: comparison in a group before and after treatment

**Supplementary Table S3. Lipid metabolites with differential changes between the dose-escalation and combination groups.**

| **m/z** | **Retention**  **Time** | **VIP** | **Metabolite** | **Adduct ion** | **Fold change** | | |
| --- | --- | --- | --- | --- | --- | --- | --- |
|  |  |  |  |  | **Values after/before in dose-escalation group** | **Values after/before in combination group** | **Values in combination/dose-escalation group after treatment** |
| **Free fatty acid** | | | | | | | |
| 255.2327 | 3.19 | 1.27 | FFA 16:0 | [M-H]- | 1.047 | 1.447^b^ | 1.330 |
| 283.2617 | 3.55 | 1.29 | FFA 18:0 | [M-H]- | 0.989 | 1.162^b^ | 1.022 |
| 281.2515 | 4.63 | 1.36 | FFA 18:1 | [M-H]- | 1.067 | 1.152^b^ | 0.998 |
| 279.2312 | 2.45 | 2.10 | FFA 18:2 | [M-H]- | 1.055 | 1.342^b^ | 1.034 |
| 389.2461 | 3.36 | 1.11 | FFA 22:5 | [M-H]- | 0.720^a^ | 0.360^b^ | 0.624 |
| **Others** | | | | | | | |
| 431.3866 | 8.59 | 1.13 | (+)-α-Tocopherol | [M+H]+ | 0.894 | 0.853^b^ | 1.083 |
| **Acylcarnitines** | | | | | | | |
| 358.2947 | 1.33 | 1.46 | Acylcarnitine 13:0 | [M]+ | 1.383 | 1.495^b^ | 1.003 |
| 372.3102 | 3.34 | 1.77 | Acylcarnitine 14:0 | [M]+ | 0.798 | 0.484^b^ | 0.716 |
| **Cholesterol esters** | | | | | | | |
| 664.5975 | 15.47 | 2.48 | CE 18:3 | [M+NH4]+ | 1.052 | 1.759^b^ | 1.759 |
| 690.6163 | 15.52 | 2.23 | CE 20:4 | [M+NH4]+ | 1.153 | 1.372^b^ | 1.220 |
| **Ceramides** | | | | | | | |
| 538.5202 | 10.72 | 1.42 | Cer 34:1 | 0.972 | 0.857 | 0.988^b^ | 0.972 |
| 622.5407 | 10.9 | 1.12 | Cer 36:2 | 1.125 | 0.753 | 0.817^b^ | 1.125 |
| 652.5868 | 14.07 | 1.22 | Cer 38:0 | 1.011 | 0.804 | 0.806^b^ | 1.011 |
| 670.594 | 14.74 | 1.91 | Cer 38:0 (2OH) | 0.978 | 0.840 | 0.800^b^ | 0.978 |
| 594.5804 | 14.08 | 2.22 | Cer 38:1 | 0.983 | 0.712 | 0.675^b^ | 0.983 |
| 682.6388 | 14.7 | 1.28 | Cer 40:0 | 0.945 | 0.863 | 0.856^b^ | 0.945 |
| 622.6104 | 14.54 | 2.87 | Cer 40:1 | 0.984 | 0.685 | 0.670^b^ | 0.984 |
| 664.6274 | 14.45 | 1.73 | Cer 40:2 | 0.900 | 0.604 | 0.648^b^ | 0.900 |
| 708.6419 | 14.47 | 1.35 | Cer 42:1 | 0.983 | 0.781 | 0.781^b^ | 0.983 |
| 706.6346 | 14.53 | 1.10 | Cer 42:2 | 1.024 | 0.810 | 0.797^b^ | 1.024 |
| 704.6195 | 14.04 | 1.13 | Cer 42:3 | 1.091 | 0.739 | 0.702^b^ | 1.091 |
| 736.6795 | 15.05 | 1.04 | Cer 44:1 | 1.061 | 0.858 | 0.832^b^ | 1.061 |
| 861.6396 | 15.06 | 1.86 | GlcCer 34:1 | 0.989 | 0.728 | 0.707^b^ | 0.989 |
| 778.6175 | 8.25 | 1.59 | GlcCer 38:1 | 1.006 | 1.096 | 1.056^b^ | 1.006 |
| 801.6808 | 14.33 | 1.41 | GlcCer 40:1 | 0.982 | 1.121 | 1.237^b^ | 0.982 |
| 814.6882 | 14.88 | 2.13 | GlcCer 42:0 | 0.981 | 0.679 | 0.685^b^ | 0.981 |
| 812.6759 | 14.9 | 1.82 | GlcCer 42:1 | 0.904 | 0.744 | 0.807^b^ | 0.904 |
| 972.7305 | 13.92 | 1.80 | LacCer 42:2 | 1.054 | 1.212 | 1.340^b^ | 1.054 |
| **Sphingomyelin** | | | | | | | |
| 697.5265 | 7.46 | 1.36 | SM 32:1 | [M+Na]+ | 0.988 | 1.122^b^ | 1.196 |
| 705.5852 | 9.69 | 2.63 | SM 34:0 | [M+H]+ | 0.966 | 1.321^b^ | 1.464 |
| 703.5718 | 9.01 | 2.17 | SM 34:1 | [M+H]+ | 0.969 | 1.194^b^ | 1.284 |
| 701.5587 | 7.6 | 1.39 | SM 34:2 | [M+H]+ | 0.990 | 1.134^b^ | 1.161 |
| 733.618 | 11.66 | 1.97 | SM 36:0 | [M+H]+ | 1.090 | 1.370^b^ | 1.624 |
| 731.6042 | 10.88 | 1.60 | SM 36:1 | [M+H]+ | 1.013 | 1.198^b^ | 1.338 |
| 787.5935 | 9.26 | 1.12 | SM 36:2 | [M+CH3COO]- | 0.993 | 1.185^b^ | 1.300 |
| 727.5705 | 7.77 | 1.50 | SM 36:3 | [M+H]+ | 1.108 | 1.197^b^ | 1.180 |
| 725.5593 | 9.01 | 2.41 | SM 36:4 | [M+Na]+ | 1.011 | 1.188^b^ | 1.221 |
| 761.5845 | 10.79 | 2.32 | SM 38:0 | [M+H]+ | 0.875 | 1.333^b^ | 1.423 |
| 757.6237 | 10.75 | 1.95 | SM 38:2 | [M+H]+ | 1.049 | 1.303^b^ | 1.378 |
| 753.5848 | 10.79 | 1.46 | SM 38:4 | [M+Na]+ | 1.053 | 1.103^b^ | 1.180 |
| 789.6776 | 14.29 | 1.45 | SM 40:0 | [M+H]+ | 0.940 | 1.203^b^ | 1.605 |
| 809.6472 | 11.14 | 1.11 | SM 40:1 | [M+Na]+ | 1.011 | 1.152^b^ | 1.239 |
| 839.693 | 14.53 | 1.62 | SM 42:0 | [M+Na]+ | 0.989 | 1.134^b^ | 1.249 |
| 873.7081 | 14.56 | 1.37 | SM 42:1 | [M+CH3COO]- | 0.963 | 1.288^b^ | 1.512 |
| 813.683 | 13.99 | 2.29 | SM 42:2 | [M+H]+ | 1.027 | 1.239^b^ | 1.298 |
| 811.6645 | 12.95 | 2.25 | SM 42:3 | [M+H]+ | 1.015 | 1.242^b^ | 1.239 |
| 899.7178 | 14.57 | 1.53 | SM 44:2 | [M+CH3COO]- | 0.998 | 1.324^b^ | 1.407 |
| 871.7386 | 13.99 | 2.06 | SM 46:1 | [M+H]+ | 0.995 | 1.470^b^ | 1.758 |
| **Diacylglycerol** | | | | | | | |
| 558.5059 | 11.88 | 2.44 | DG 30:0 | [M+NH4]+ | 0.822 | 0.402^b^ | 0.526 |
| 556.4974 | 9.95 | 2.48 | DG 30:1 | [M+NH4]+ | 0.951 | 0.388^b^ | 0.391 |
| 586.5385 | 13.69 | 2.62 | DG 32:0 | [M+NH4]+ | 0.719^a^ | 0.488^b^ | 0.686 |
| 584.5244 | 11.99 | 2.38 | DG 32:1 | [M+NH4]+ | 0.754 | 0.525^b^ | 0.645 |
| 582.5058 | 10.3 | 3.08 | DG 32:2 | [M+NH4]+ | 0.834 | 0.416^b^ | 0.474 |
| 614.5693 | 14.35 | 2.58 | DG 34:0 | [M+NH4]+ | 0.834 | 0.564^b^ | 0.709 |
| 595.5276 | 13.68 | 2.45 | DG 34:1 | [M+H]+ | 0.776^a^ | 0.615^b^ | 0.746 |
| 610.5365 | 12.44 | 3.19 | DG 34:2 | [M+NH4]+ | 0.799 | 0.470^b^ | 0.593 |
| 608.5195 | 10.43 | 2.20 | DG 34:3 | [M+NH4]+ | 0.912 | 0.564^b^ | 0.628 |
| 640.5904 | 14.38 | 2.83 | DG 36:1 | [M+NH4]+ | 0.821 | 0.482^b^ | 0.579 |
| 621.5443 | 13.71 | 2.44 | DG 36:2 | [M+H]+ | 0.949 | 0.684^b^ | 0.647 |
| 636.5567 | 12.54 | 2.68 | DG 36:3 | [M+NH4] | 0.943 | 0.483^b^ | 0.502 |
| 634.5395 | 10.87 | 2.70 | DG 36:4 | [M+H]+ | 0.967 | 0.373^b^ | 0.373 |
| 615.4916 | 10.44 | 3.02 | DG 36:5 | [M+H]+ | 0.881 | 0.739^b^ | 0.809 |
| 664.5823 | 14.84 | 2.69 | DG 38:3 | [M+NH4]+ | 0.972 | 0.590^b^ | 0.543 |
| 645.5394 | 14.37 | 2.95 | DG 38:4 | [M+H]+ | 0.949 | 0.768^b^ | 0.808 |
| 643.526 | 13.74 | 2.28 | DG 38:5 | [M+H]+ | 0.946 | 0.672^b^ | 0.682 |
| 641.5048 | 12.51 | 2.78 | DG 38:6 | [M+H] | 0.931 | 0.522^b^ | 0.552 |
| 656.5188 | 9.26 | 2.28 | DG 38:7 | [M+NH4]+ | 0.946 | 0.520^b^ | 0.645 |
| 694.6853 | 14.72 | 2.47 | DG 40:2 | [M+NH4]+ | 1.004 | 0.778^b^ | 0.721 |
| 686.5838 | 14.5 | 2.23 | DG 40:6 | [M+NH4]+ | 1.029 | 0.743^b^ | 0.740 |
| 684.5554 | 11.42 | 1.45 | DG 40:7 | [M+NH4]+ | 0.959 | 0.624^b^ | 0.740 |
| 682.5349 | 9.9 | 1.87 | DG 40:8 | [M+NH4]+ | 0.944 | 0.491^b^ | 0.598 |
| **Lysophosphatidylglycerol** | | | | | | | |
| 437.2432 | 2.99 | 1.07 | LysoPA 17:0 | [M+FA-H]- | 0.723 | 0.366^b^ | 0.698 |
| 465.3025 | 4.75 | 1.02 | LysoPA 20:0 | [M-H]- | 0.893 | 1.239^b^ | 1.363 |
| 518.2992 | 2.44 | 1.63 | LysoPC 16:0 | [M+Na]+ | 1.535 | 0.942^b^ | 1.505 |
| 494.3218 | 2.08 | 1.88 | LysoPC 16:1 | [M+H]+ | 0.932 | 1.460^b^ | 1.542 |
| 524.3687 | 3.93 | 1.01 | LysoPC 18:0 | [M+H]+ | 0.907 | 0.849^b^ | 0.887 |
| 522.3539 | 3 | 1.79 | LysoPC 18:1 | [M+H]+ | 0.962 | 1.292^b^ | 1.216 |
| 518.3233 | 1.85 | 1.63 | LysoPC 18:3 | [M+H]+ | 0.942 | 1.535^b^ | 1.505 |
| 516.302 | 2.09 | 1.89 | LysoPC 18:4 | [M+H]+ | 0.911 | 1.49^b^ | 1.598 |
| 610.4099 | 5.05 | 1.36 | LysoPC 20:0 | [M+CH3COO]- | 0.841 | 0.785^b^ | 0.826 |
| 544.3354 | 2.99 | 1.85 | LysoPC 20:4 | [M+H]+ | 0.968 | 1.255^b^ | 1.194 |
| 580.4055 | 2.99 | 1.08 | LysoPC 22:0 | [M+H]+ | 0.819 | 1.299^b^ | 1.318 |
| 568.3399 | 2.07 | 1.49 | LysoPC 22:6 | [M+H]+ | 0.929 | 0.804^b^ | 0.842 |
| 480.3059 | 3.13 | 1.29 | LysoPE 18:1 | [M+H]+ | 0.955 | 1.259^b^ | 1.149 |
| 478.291 | 2.41 | 1.41 | LysoPE 18:2 | [M+H]+ | 0.893 | 1.277^b^ | 1.265 |
| 504.3069 | 2.7 | 1.95 | LysoPE 20:3 | [M+H]+ | 1.017 | 1.228^b^ | 1.187 |
| 502.2874 | 2.32 | 1.15 | LysoPE 20:4 | [M+H]+ | 1.031 | 1.214^b^ | 1.124 |
| **Phosphatidylamine** | | | | | | | |
| 771.5254 | 9.09 | 1.13 | PA 38:3 | [M+FA-H]- | 1.318 | 0.494^b^ | 0.445 |
| **Phosphatidylcholine** | | | | | | | |
| 704.5199 | 7.5 | 1.30 | PC 30:1 | [M+H]+ | 0.861 | 1.420^b^ | 1.595 |
| 732.5532 | 9.03 | 1.94 | PC 32:1 | [M+H]+ | 0.852 | 1.570^b^ | 1.679 |
| 730.5338 | 7.77 | 2.23 | PC 32:2 | [M+H]+ | 0.976 | 0.739^b^ | 0.753 |
| 762.5977 | 12.8 | 1.88 | PC 34:0 | [M+H]+ | 0.894 | 0.860^b^ | 0.958 |
| 760.584 | 10.83 | 2.41 | PC 34:1 | [M+H]+ | 0.882 | 1.444^b^ | 1.527 |
| 758.5673 | 9.38 | 1.03 | PC 34:2 | [M+H]+ | 0.937 | 0.886^b^ | 0.938 |
| 778.5276 | 8.56 | 2.03 | PC 34:3 | [M+Na]+ | 0.942 | 1.445^b^ | 1.394 |
| 771.5728 | 10.81 | 2.72 | PC 34:4 | [M+H]+ | 0.872 | 1.483^b^ | 1.568 |
| 752.5217 | 6.82 | 2.22 | PC 34:5 | [M+H]+ | 1.137 | 0.606^b^ | 0.529 |
| 848.6861 | 14.33 | 1.45 | PC 36:0 | [M+CH3COO]- | 0.889 | 1.346^b^ | 1.725 |
| 788.6088 | 12.93 | 2.30 | PC 36:1 | [M+H]+ | 0.880 | 1.511^b^ | 1.556 |
| 786.5983 | 11.26 | 1.11 | PC 36:2 | [M+H]+ | 0.950 | 0.851^b^ | 0.865 |
| 784.5817 | 10.46 | 2.51 | PC 36:3 | [M+H]+ | 0.979 | 2.077^b^ | 1.899 |
| 804.545 | 7.41 | 1.62 | PC 36:4 | [M+Na]+ | 1.080 | 0.864^b^ | 0.865 |
| 778.5339 | 7.27 | 3.12 | PC 36:6 | [M+H]+ | 0.996 | 0.531^b^ | 0.550 |
| 776.5786 | 8.81 | 2.41 | PC 36:7 | [M+H]+ | 0.941 | 1.437^b^ | 1.530 |
| 818.6354 | 10.73 | 2.45 | PC 38:0 | [M+H]+ | 0.933 | 1.186^b^ | 1.279 |
| 816.619 | 9.09 | 1.53 | PC 38:1 | [M+H]+ | 1.015 | 1.236^b^ | 1.276 |
| 814.6324 | 13.15 | 2.12 | PC 38:2 | [M+H]+ | 0.886 | 1.260^b^ | 1.273 |
| 812.6127 | 12.53 | 2.53 | PC 38:3 | [M+H]+ | 0.942 | 2.471^b^ | 2.156 |
| 832.5753 | 10.37 | 1.09 | PC 38:4 | [M+Na]+ | 0.988 | 1.142^b^ | 1.096 |
| 808.5844 | 9.2 | 1.68 | PC 38:5 | [M+H]+ | 0.979 | 0.842^b^ | 0.885 |
| 806.5659 | 8.68 | 2.34 | PC 38:6 | [M+H]+ | 0.861 | 0.693^b^ | 0.849 |
| 826.5666 | 13 | 2.34 | PC 38:7 | [M+H]+ | 0.880 | 1.507^b^ | 1.590 |
| 802.6364 | 11.74 | 1.93 | PC 38:8 | [M+H]+ | 0.939 | 1.274^b^ | 1.519 |
| 817.5604 | 8.69 | 2.26 | PC 38:9 | [M+H]+ | 0.878 | 0.673^b^ | 0.814 |
| 844.6522 | 11.27 | 1.18 | PC 40:1 | [M+H]+ | 0.962 | 0.865^b^ | 0.886 |
| 842.6323 | 10.45 | 2.08 | PC 40:2 | [M+H]+ | 0.934 | 1.862^b^ | 1.800 |
| 894.6147 | 10.49 | 1.08 | PC 40:5 | [M+CH3COO]- | 0.890 | 0.843^b^ | 0.952 |
| 834.6002 | 10.41 | 1.72 | PC 40:6 | [M+H]+ | 0.878 | 0.767^b^ | 0.905 |
| 832.5835 | 8.74 | 1.77 | PC 40:7 | [M+H]+ | 1.013 | 0.790^b^ | 0.735 |
| 830.5674 | 7.63 | 1.72 | PC 40:8 | [M+H]+ | 0.939 | 0.865^b^ | 0.824 |
| 854.5749 | 8.7 | 1.95 | PC 40:10 | [M+H]+ | 0.868 | 1.294^b^ | 1.413 |
| 870.7163 | 12.98 | 2.18 | PC 42:2 | [M+H]+ | 0.933 | 1.511^b^ | 1.605 |
| 866.635 | 9.21 | 2.06 | PC 42:4 | [M+H]+ | 0.960 | 0.865^b^ | 0.939 |
| 864.6225 | 8.72 | 2.37 | PC 42:5 | [M+H]+ | 0.883 | 0.737^b^ | 0.877 |
| 852.563 | 7.58 | 1.56 | PC 42:11 | [M+H]+ | 0.789^a^ | 1.224^b^ | 1.556 |
| 894.6665 | 11.02 | 1.51 | PC 44:4 | [M+H]+ | 0.879 | 0.801^b^ | 0.942 |
| 892.6495 | 10.41 | 1.54 | PC 44:5 | [M+H]+ | 0.810 | 0.728^b^ | 0.921 |
| 890.6369 | 8.77 | 1.71 | PC 44:6 | [M+H]+ | 0.994 | 0.810^b^ | 0.784 |
| 1002.8163 | 15.15 | 1.11 | PC 48:0 | [M+FA-H]- | 1.014 | 1.179^b^ | 1.164 |
| **Phosphatidylethanolamine** | | | | | | | |
| 740.522 | 9.46 | 1.30 | PE 36:4 | [M+H]+ | 0.946 | 1.223^b^ | 1.356 |
| 764.5179 | 9.02 | 1.31 | PE 38:5 | [M+NH4]+ | 0.903 | 0.858^b^ | 1.118 |
| 794.5639 | 12.24 | 1.25 | PE 40:5 | [M+H]+ | 0.879 | 0.815^b^ | 0.993 |
| 792.5472 | 10.83 | 2.11 | PE 40:6 | [M+NH4]+ | 0.913 | 0.737^b^ | 0.984 |
| **Phosphatidylinositol** | | | | | | | |
| 807.5059 | 7.3 | 1.46 | PI 32:1 | [M-H]- | 0.559^a^ | 2.228^b^ | 3.427 |
| 835.5408 | 8.62 | 1.18 | PI 34:1 | [M-H]- | 0.701^a^ | 1.553^b^ | 2.075 |
| 859.5447 | 8.3 | 1.65 | PI 36:3 | [M-H]- | 0.745 | 1.448^b^ | 1.988 |
| 857.5261 | 7.41 | 1.20 | PI 36:4 | [M-H]- | 0.726^a^ | 1.422^b^ | 1.641 |
| 887.5739 | 9.42 | 1.56 | PI 38:3 | [M-H]- | 0.901 | 0.752^b^ | 0.835 |
| 911.574 | 8.79 | 1.28 | PI 40:5 | [M-H]- | 0.854 | 0.774^b^ | 1.057 |
| **Triacylglycerol** | | | | | | | |
| 656.5806 | 14.36 | 1.38 | TG 36:0 | [M+NH4]+ | 1.134 | 0.803^b^ | 0.769 |
| 712.6384 | 15.01 | 1.36 | TG 40:0 | [M+NH4]+ | 0.952 | 0.557^b^ | 0.545 |
| 740.6756 | 15.22 | 1.80 | TG 42:0 | [M+NH4]+ | 0.981 | 0.550^b^ | 0.626 |
| 738.6566 | 15 | 1.85 | TG 42:1 | [M+NH4]+ | 0.730^a^ | 0.390^b^ | 0.383 |
| 736.6423 | 14.77 | 1.69 | TG 42:2 | [M+NH4]+ | 0.846 | 0.540^b^ | 0.443 |
| 768.7005 | 15.38 | 1.96 | TG 44:0 | [M+NH4]+ | 0.950 | 0.725^b^ | 0.765 |
| 766.6894 | 15.22 | 1.97 | TG 44:1 | [M+NH4]+ | 0.869 | 0.483^b^ | 0.568 |
| 762.6625 | 14.86 | 2.47 | TG 44:3 | [M+NH4]+ | 0.813 | 0.468^b^ | 0.514 |
| 743.615 | 14.99 | 1.72 | TG 44:4 | [M+H]+ | 0.691 | 0.447^b^ | 0.438 |
| 741.5965 | 14.74 | 1.57 | TG 44:5 | [M+H]+ | 0.838 | 0.582^b^ | 0.480 |
| 794.7235 | 15.38 | 1.92 | TG 46:1 | [M+NH4]+ | 0.853 | 0.586^b^ | 0.654 |
| 792.7082 | 15.23 | 2.10 | TG 46:2 | [M+NH4]+ | 0.906 | 0.565^b^ | 0.632 |
| 790.6865 | 15.06 | 2.52 | TG 46:3 | [M+NH4]+ | 0.880 | 0.513^b^ | 0.577 |
| 771.6432 | 15.19 | 1.81 | TG 46:4 | [M+H]+ | 0.870 | 0.519^b^ | 0.598 |
| 769.6223 | 15.03 | 2.00 | TG 46:5 | [M+NH4]+ | 0.798 | 0.542^b^ | 0.619 |
| 820.7363 | 15.37 | 1.43 | TG 48:2 | [M+NH4]+ | 0.832 | 0.750^b^ | 0.834 |
| 818.7181 | 15.21 | 2.41 | TG 48:3 | [M+NH4]+ | 0.912 | 0.630^b^ | 0.679 |
| 799.6712 | 15.38 | 1.78 | TG 48:4 | [M+H]+ | 0.864 | 0.641^b^ | 0.707 |
| 797.6612 | 15.24 | 2.05 | TG 48:5 | [M+H]+ | 0.903 | 0.615^b^ | 0.663 |
| 835.7508 | 15.4 | 1.79 | TG 50:0 | [M+NH4]+ | 0.895 | 0.721^b^ | 0.760 |
| 846.7534 | 15.38 | 1.60 | TG 50:3 | [M+NH4]+ | 0.936 | 0.824^b^ | 0.858 |
| 844.7353 | 15.26 | 2.07 | TG 50:4 | [M+NH4]+ | 0.950 | 0.727^b^ | 0.732 |
| 842.7203 | 15.09 | 2.00 | TG 50:5 | [M+NH4]+ | 0.991 | 0.725^b^ | 0.709 |
| 819.6579 | 14.04 | 1.35 | TG 50:8 | [M+NH4]+ | 0.994 | 1.183^b^ | 1.250 |
| 885.7621 | 15.24 | 1.98 | TG 52:0 | [M+Na]+ | 1.018 | 0.768^b^ | 0.733 |
| 878.7968 | 15.58 | 1.37 | TG 52:1 | [M+NH4]+ | 0.981 | 0.894^b^ | 0.839 |
| 859.7498 | 15.22 | 2.60 | TG 52:2 | [M+NH4]+ | 0.957 | 0.668^b^ | 0.693 |
| 874.7843 | 15.52 | 1.34 | TG 52:3 | [M+NH4]+ | 1.005 | 0.864^b^ | 0.849 |
| 872.7691 | 15.41 | 1.75 | TG 52:4 | [M+NH4]+ | 1.039 | 0.774^b^ | 0.727 |
| 870.7511 | 15.27 | 1.59 | TG 52:5 | [M+NH4]+ | 1.108 | 0.799^b^ | 0.730 |
| 851.7025 | 15.36 | 1.37 | TG 52:6 | [M+H]+ | 0.932 | 0.876^b^ | 0.905 |
| 849.691 | 15.26 | 1.97 | TG 52:7 | [M+H]+ | 0.952 | 0.763^b^ | 0.766 |
| 900.7946 | 15.51 | 1.52 | TG 54:4 | [M+NH4]+ | 1.178 | 0.801^b^ | 0.694 |
| 879.7343 | 15.51 | 1.73 | TG 54:6 | [M+H]+ | 1.008 | 0.826^b^ | 0.817 |
| 877.7216 | 15.39 | 1.79 | TG 54:7 | [M+H]+ | 1.022 | 0.775^b^ | 0.743 |
| 875.7067 | 15.29 | 1.61 | TG 54:8 | [M+NH4]+ | 1.075 | 0.811^b^ | 0.755 |
| 913.7984 | 15.41 | 1.67 | TG 56:3 | [M+H]+ | 1.052 | 0.793^b^ | 0.746 |
| 907.7701 | 15.61 | 1.60 | TG 56:6 | [M+H]+ | 1.135 | 0.834^b^ | 0.737 |
| 905.7554 | 15.51 | 1.63 | TG 56:7 | [M+H]+ | 1.160 | 0.799^b^ | 0.712 |
| 917.6677 | 13.94 | 2.24 | TG 56:12 | [M+Na]+ | 1.019 | 1.192^b^ | 1.224 |
| 919.6832 | 14.54 | 1.66 | TG 58:14 | [M+H]+ | 0.985 | 1.165^b^ | 1.266 |
| 1002.8313 | 13.71 | 2.64 | TG 62:9 | [M+NH4]+ | 0.788 | 0.569^b^ | 0.668 |
| 981.7642 | 13.69 | 2.40 | TG 62:11 | [M+H]+ | 0.710^a^ | 0.519^b^ | 0.748 |
| 1028.8433 | 13.74 | 2.41 | TG 64:10 | [M+NH4]+ | 0.976 | 0.648^b^ | 0.625 |
| 1026.8343 | 12.55 | 2.57 | TG 64:11 | [M+NH4]+ | 0.984 | 0.496^b^ | 0.494 |
| 1007.7817 | 13.71 | 2.74 | TG 64:12 | [M+H]+ | 0.818 | 0.585^b^ | 0.713 |
| 1035.8145 | 14.35 | 2.74 | TG 66:12 | [M+H]+ | 0.862 | 0.581^b^ | 0.670 |
| 1023.7654 | 13.73 | 2.73 | TG 66:18 | [M+H]+ | 0.832 | 0.614^b^ | 0.708 |

^a^:*p* value<0.05 metabolites between before and after dose escalation therapy

^b^:*p* value<0.05 metabolites between before and after combination therapy

All *p*-values were adjusted by false discovery rate method.

**Supplementary Table S4. Aqueous metabolites showing differential changes between the dose-escalation and combination groups.**

| **m/z** | **Retention time** | **VIP** | **Metabolite** | **Adduct ion** | **Fold change** | | |
| --- | --- | --- | --- | --- | --- | --- | --- |
|  |  |  |  |  | **Values after/before in dose-escalation group** | **Values after/before in combination group** | **Values in combination/**  **dose-escalation group after treatment** |
| **Fatty acids (5)** | | | | | | | |
| 327.2315 | 0.66 | 1.46 | Docosahexaenoic acid* | [M+H]+ | 0.96 | 0.57^b^ | 0.65 |
| 211.135 | 0.76 | 1.58 | Dodecanedioic acid | [M-H]- | 1.22 | 0.64^b^ | 0.58 |
| 271.2268 | 0.76 | 2.02 | 2-hydroxyhexadecanoic acid | [M-H]- | 0.98 | 0.51^b^ | 0.56 |
| 274.2718 | 0.54 | 1.27 | Palmitic acid* | [M+H]+ | 0.52 | 0.77^b^ | 0.93 |
| 288.2878 | 2.92 | 1.57 | Margaric acid | [M+H]+ | 1.04 | 0.78^b^ | 0.86 |
| 289.1168 | 1.13 | 1.26 | Pentadecanoic acid, | [M-H]- | 1.05 | 0.69^b^ | 0.74 |
| **Organic acids (14)** | | | | | | | |
| 145.0506 | 2.03 | 1.4 | Adipic acid* | [M-H]- | 0.69 | 0.39^b^ | 0.74 |
| 135.0304 | 8.95 | 1.18 | Glycolic acid* | [M-H]- | 1.07 | 0.72^b^ | 0.84 |
| 195.052 | 9.65 | 1.44 | Gluconic acid | [M-H]- | 0.76 | 0.78^b^ | 0.88 |
| 186.0578 | 6.03 | 1.47 | Indolelactic acid* | [M-H]- | 0.96 | 0.73^b^ | 0.81 |
| 130.0847 | 6.64 | 1.81 | Nipecotic acid | [M+H]+ | 1.77 | 0.36^b^ | 0.44 |
| 220.1164 | 2.32 | 1.42 | Pantothenic acid* | [M+H]+ | 0.88 | 1.40^b^ | 1.73 |
| 117.0208 | 5.01 | 1.31 | Succinic acid* | [M-H]- | 1.06 | 0.74^b^ | 0.81 |
| 160.1308 | 5.18 | 1.85 | DL-2-Aminooctanoic acid | [M+H]+ | 1.31 | 0.59^b^ | 0.51 |
| 191.0191 | 9.56 | 1.07 | Citric acid* | [M-H]- | 1.23 | 0.83^b^ | 0.80 |
| 331.1926 | 0.75 | 1.57 | Carnosic acid* | [M-H]- | 0.79 | 0.63^b^ | 0.75 |
| 130.0884 | 6.22 | 1.20 | Aminocaproic acid | [M-H]- | 1.02 | 0.77^b^ | 0.77 |
| 190.0533 | 5.84 | 1.47 | 5-Hydroxyindoleacetic acid | [M-H]- | 1.00 | 0.82^b^ | 0.90 |
| 131.0708 | 2.4 | 1.07 | Leucinic acid* | [M-H]- | 1.04 | 0.65^b^ | 0.91 |
| 145.0529 | 1.19 | 1.56 | 2,2-Dimethyl Succinic acid | [M-H]- | 1.00 | 0.76^b^ | 0.79 |
| **Carnitine and derivatives (5)** | | | | | | | |
| 246.1696 | 5.64 | 1.83 | 2-Methylbutyroylcarnitine | [M+H]+ | 0.91 | 1.58^b^ | 1.30 |
| 305.2475 | 4.96 | 2.06 | Octanoyl-carnitine* | [M+H]+ | 1.53 | 1.92^b^ | 1.03 |
| 162.1123 | 7.79 | 2.37 | L-Carnitine* | [M+H]+ | 0.78 | 4.16^b^ | 4.97 |
| 204.122 | 7.66 | 1.74 | Acetyl-carnitine* | [M+H]+ | 1.19^a^ | 1.21^b^ | 1.09 |
| 146.1163 | 8.36 | 1.07 | Acetylcholine* | [M+H]+ | 1.19 | 2.40^b^ | 2.75 |
| **Bile acids(3)** | | | | | | | |
| 391.2853 | 0.81 | 1.48 | Deoxycholic acid* | [M-H]- | 1.05 | 0.66^b^ | 0.69 |
| 453.2895 | 0.66 | 1.43 | Cholic acid or Allocholic acid* | [M-H]- | 0.94 | 0.65^b^ | 0.75 |
| 160.1329 | 7.59 | 2.27 | Metacholine | [M+H]+ | 1.09 | 1.93^b^ | 1.76 |
| **Purine derivatives (2)** | | | | | | | |
| 135.0321 | 3.34 | 1.09 | Hypoxanthine* | [M-H]- | 0.94 | 0.69^b^ | 0.89 |
| 167.0219 | 6.98 | 2.16 | Uric acid* | [M-H]- | 0.98 | 0.70^b^ | 0.70 |
| **Amino acids (11)** | | | | | | | |
| 164.0727 | 5.79 | 1.14 | DL-Phenylalanine* | [M-H]- | 0.98 | 0.87^b^ | 0.89 |
| 241.0313 | 9.46 | 1.54 | L-Cystine* | [M+H]+ | 1.26 | 1.37^b^ | 1.19 |
| 145.0627 | 8.01 | 1.35 | L-Glutamine* | [M-H]- | 1.04 | 0.84^b^ | 0.84 |
| 154.0629 | 9.73 | 1.03 | L-Histidine* | [M-H]- | 0.97 | 0.88^b^ | 0.89 |
| 150.0571 | 6.63 | 1.64 | L-Methionine* | [M+H]+ | 1.06 | 1.29^b^ | 1.25 |
| 130.0881 | 5.98 | 1.20 | L-Norleucine* | [M-H]- | 1.01 | 0.82^b^ | 0.80 |
| 180.0677 | 7.06 | 1.00 | L-Tyrosine* | [M-H]- | 0.97 | 0.88^b^ | 0.94 |
| 118.0861 | 8.13 | 1.47 | L-Valine* | [M+H]+ | 1.01 | 0.92^b^ | 0.95 |
| 207.0798 | 5.84 | 1.25 | Kynurenine* | [M-H]- | 1.01 | 0.85^b^ | 0.90 |
| 203.0826 | 6.02 | 1.59 | Tryptophan* | [M-H]- | 0.96 | 0.77^b^ | 0.80 |
| 152.068 | 6.85 | 1.99 | 2-Phenylglycine* | [M+H]+ | 0.59 | 3.91^b^ | 6.67 |
| **Others (2)** | | | | | | | |
| 121.0284 | 0.74 | 1.55 | 4-Hydroxybenzaldehyde | [M-H]- | 0.95 | 0.79^b^ | 0.82 |
| 132.0756 | 7.73 | 1.39 | Creatine* | [M+H]+ | 0.96 | 1.46^b^ | 1.28 |
| **Drugs (2)** | | | | | | | |
| 194.1162 | 7.45 | 1.59 | MDMA | [M+H]+ | 1.05 | 0.89^b^ | 0.90 |
| 317.057 | 1.23 | 2.69 | Fenofibric acid | [M-H]- | 19.91 | 722.92^b^ | 20.64 |

*: confirmed by analytical standards

^a^:*p* value<0.05 metabolites between before and after dose escalation therapy

^b^:*p* value<0.05 metabolites between before and after combination therapy

All *p*-values were adjusted by false discovery rate method.
